# Engineering of soybean mosaic virus as a versatile tool for studying protein–protein interactions in soybean

**DOI:** 10.1038/srep22436

**Published:** 2016-02-29

**Authors:** Jang-Kyun Seo, Hong-Soo Choi, Kook-Hyung Kim

**Affiliations:** 1 Crop Protection Division, National Academy of Agricultural Science, Rural Development Administration, Wanju 565-851, Republic of Korea; 2 Department of Agricultural Biotechnology and Plant Genomics and Breeding Institute, Seoul National University, Seoul 151-921, Republic of Korea

## Abstract

Transient gene expression approaches are valuable tools for rapid introduction of genes of interest and characterization of their functions in plants. Although agroinfiltration is the most effectively and routinely used method for transient expression of multiple genes in various plant species, this approach has been largely unsuccessful in soybean. In this study, we engineered soybean mosaic virus (SMV) as a dual-gene delivery vector to simultaneously deliver and express two genes in soybean cells. We further show the application of the SMV-based dual vector for a bimolecular fluorescence complementation assay to visualize *in vivo* protein–protein interactions in soybean and for a co-immunoprecipitation assay to identify cellular proteins interacting with SMV helper component protease. This approach provides a rapid and cost-effective tool for transient introduction of multiple traits into soybean and for *in vivo* characterization of the soybean cellular protein interaction network.

Transient gene expression systems, including *Agrobacterium*-mediated gene delivery (agroinfiltration), particle bombardment, and virus-based gene expression and silencing vectors, have proven to be powerful and versatile tools for gain-of-function and loss-of-function approaches in plant molecular, cellular, genetic, and proteomic studies. In particular, agroinfiltration has been used widely and effectively for ectopic expression of genes of interest and examination of their functions^1^,^2^. Most importantly, agroinfiltration facilitates synchronized delivery of multiple transgenes into the same cell, offering the advantage of expressing multiple recombinant proteins in the same cell and examining their interactions and functions^3^,^4^. However, despite efficient application of agroinfiltration in various plant systems, this method has been largely unsuccessful in soybean.

Alternatively, virus-mediated gene delivery systems have been developed for systemic expression of recombinant proteins and gene silencing in soybean plants^5^,^6^,^7^. Virus-mediated expression systems are superior to other transient gene expression systems as well as the transgenic approach because viruses infect their host plants systemically and replicate to high titers, so that large amounts of recombinant proteins accumulate within a short period of time^8^. To date, a few viruses, including bean pod mottle virus (BPMV), cucumber mosaic virus (CMV), and soybean mosaic virus (SMV), have been engineered as gene delivery vectors for systemic expression of recombinant proteins in soybean^5^,^7^,^9^. However, the viral vectors derived from multipartite viruses (i.e., BPMV and CMV) have some restrictions in introducing and expressing desired genes in plants because the estimated maximum DNA fragment sizes that can be inserted into BPMV and CMV genomes are approximately 1.8 kb and 0.9 kb, respectively^5^. In addition, expensive and complicated inoculation procedures, such as particle bombardment and *in vitro* transcription, are required to achieve high infection rates with these multipartite virus-derived vectors.

SMV, which has a monopartite single-stranded RNA genome of approximately 9.6 kb, is a member of the genus *Potyvirus*. Various potyviruses have been developed as viral gene delivery vectors^8^. Advantageous features of potyvirus-mediated expression systems include simultaneous equimolecular expression of multiple desired genes and relatively flexible length of the foreign genes (up to 4 kb) that can be expressed^10^,^11^. In our previous study, we developed an SMV-based gene delivery vector by engineering the cloning sites and the additional NIa-Pro cleavage site between SMV P1 and helper component protease (HC-Pro) cistrons and successfully expressed single recombinant proteins in soybean^7^. As in other potyvirus-mediated expression systems^10^, recombinant proteins expressed by the SMV-based vector are synthesized as part of the viral polyprotein. We also showed that simple rub-inoculation of plasmid DNAs of the SMV-based viral vectors was successful to cause infection and systemically express recombinant proteins in soybean plants^7^.

In the present study, we further engineered SMV as a dual-gene delivery vector to simultaneously deliver and express two genes in soybean cells. We successfully visualized subcellular localization of two different fluorescent proteins in soybean cells using the SMV-based dual vector. In addition, we applied the SMV-based dual vector system to a bimolecular fluorescence complementation (BiFC) assay to visualize *in vivo* protein–protein interactions in soybean. We described the detailed procedure for a co-immunoprecipitation (co-IP) assay in combination with the SMV-based dual vector to identify cellular proteins interacting with SMV HC-Pro. We expect that our procedures will provide useful tools to the soybean research community.

## Results and Discussion

### Engineering of SMV as a dual-gene delivery vector

We previously developed a promising gene delivery system by engineering the full-length infectious cDNA clone of SMV strain G7H (pSMV-G7H)^7^. The viral vector, named pSMV-MCS, contains a single gene insertion cassette between P1 and HC-Pro^7^. The desired genes can be stably and systemically delivered into soybean by simple rub-inoculation with intact plasmid DNA of this recombinant SMV-based vector.

**Figure 1:**
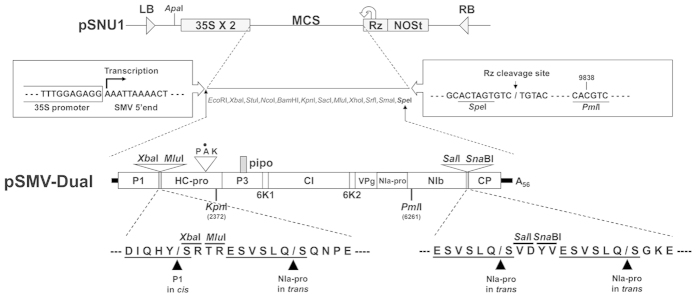
Schematic representation of the construction of pSMV-Dual vector. The binary vector, pSNU1, contains, in sequential order, a left border of T-DNA (LB), a double 35S promoter (35S), multiple cloning site (MCS), a cis-cleaving ribozyme sequence (Rz), a NOS terminator (NOSt), and a right border of T-DNA (RB). pSMV-Dual contains two gene insertion cassettes between the P1 and HC-Pro cistron and between the NIb and CP cistrons. Each gene insertion cassette contains two unique cloning sites as indicated. Amino acid sequences of the peptide cleavage sites recognized by either the P1 or NIa-Pro viral proteases are underlined and arrowheads indicate the location of the cleaved peptide bond. A non-aphid-transmissible mutation (substitution of Thr to Ala in the PTK motif of HC-Pro) was introduced into pSMV-Dual.

In the current study, we modified pSMV-MCS by engineering an additional gene insertion cassette between nuclear inclusion b (NIb) and coat protein (CP) cistrons. The second gene insertion cassette is composed of two restriction enzyme sites (*Sal*I and *Sna*BI) and an additional NIa-Pro cleavage site (ESVSLQ) (Fig. 1). To minimize the potential for homologous recombination during plasmid DNA replication and subsequent instability, each residue of the inserted NIa-Pro cleavage site was selected based on the codon usage frequency for SMV. Abolishment of plant-to-plant transmission capacity of a plant virus-based vector is an important environmental issue that may facilitate its field application. Therefore, a non-aphid-transmissible mutation (substitution of Thr to Ala at amino acid position 310 of SMV HC-Pro; T310A) was introduced into the P_309_T_310_K_311_ motif of HC-Pro, which is the critical motif for aphid transmission of potyviruses^12^. The resulting dual-gene delivery vector was named pSMV-Dual (Fig. 1). Two different genes can be delivered simultaneously by the pSMV-Dual vector upon utilizing two gene insertion cassettes to create an in-frame translational fusion. Therefore, the recombinant protein expressed from the first gene insertion cassette will have two additional amino acids (SR) at the N-terminus and 10 amino acids (SRTRESVSLQ) at the C-terminus when cloned by utilizing the *Xba*I site. Proteolysis will produce a recombinant protein having three (SVD) and 10 (VDYVESVSLQ) additional amino acids at the N-terminus and C-terminus, respectively, when cloned by utilizing the *Sal*I site (Fig. 1).

In the previous study, we showed that DNA-mediated rub-inoculation of the SMV infectious cDNA construct yielded highly efficient infection on soybean plants^7^. Thus, in the present study, we sought to examine whether the additional insertion of the gene cassette between the NIb and CP cistrons affects the infectivity of the pSMV-Dual plasmid. To this end, soybean seedlings (cv. Lee74, used throughout this study) were rub-inoculated with different quantities (10 μg, 5 μg, or 1 μg) of plasmid DNA of pSMV-Dual. Experiments were carried out three times independently, using 45 plants in total (Table 1). Infection of the inoculated plants with SMV was investigated by observing the appearance of symptoms on systemic leaves and by RT-PCR analysis using SMV-specific primers as described previously^7^. At 15 days post inoculation (dpi), all of the soybean plants inoculated with 10 μg, 5 μg, or 1 μg of plasmid DNA showed typical mild mosaic symptoms in upper uninoculated leaves. RT-PCR analysis further confirmed that all the inoculated plants were systemically infected (Table 1).

To confirm the effect of the introduced non-aphid-transmissible mutation into pSMV-Dual, we conducted a plant-to-plant transmission assay using aphids (*Aphis glycines*). The transmission assays were performed three times independently, comprising 15 plants for each construct (Table 2). Although the progeny viruses of pSMV-G7H and -MCS were successfully transmitted, no aphid transmission of the progeny viruses of pSMV-Dual was observed, demonstrating that the introduced mutation abolished aphid transmissibility of pSMV-Dual.

### Simultaneous expression of two recombinant proteins and visualization of their subcellular accumulation

**Figure 2:**
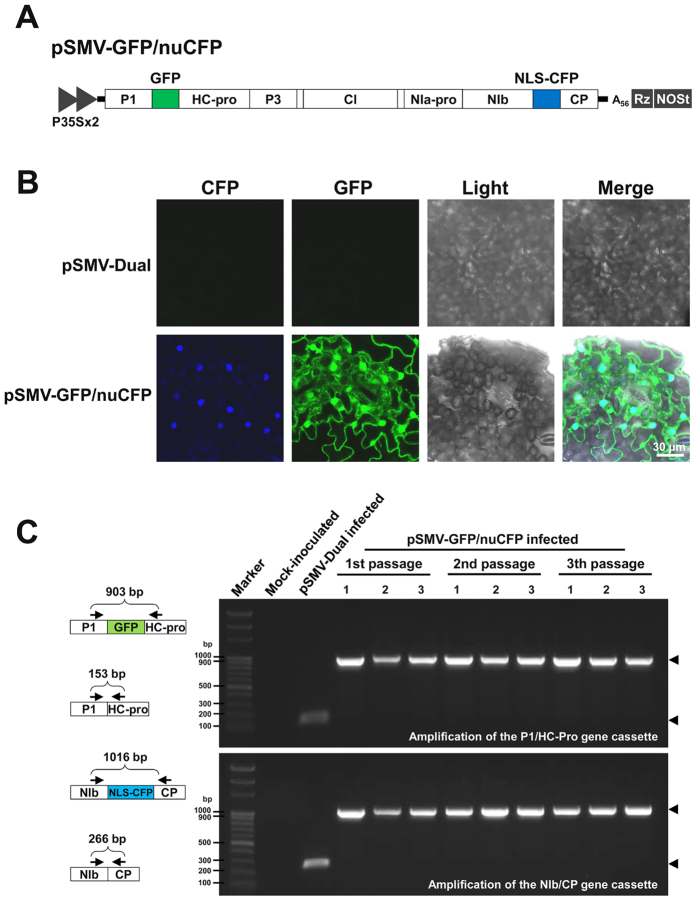
Simultaneous expression and visualization of two recombinant proteins in soybean cells using the SMV-based dual vector (**A**) SMV genome organization and simultaneous insertion of the *gfp* gene between P1 and HC-Pro and the NLS-tagged *cfp* gene between NIb and CP. (**B**) Confocal images showing subcellular distribution of GFP and NLS-tagged CFP expressed in soybean cells by the SMV-based dual vector. No fluorescence was detected in the soybean cells infected by pSMV-Dual. (**C**) Analysis of stability of heterologous gene insertions in the SMV genome. Schematic maps of SMV P1/HC-Pro, P1/GFP/HC-Pro, NIb/CP, and NIb/NLS-CFP/CP regions are shown. Arrows indicate the regions that have been amplified. Total RNAs, isolated from mock inoculated, pSMV-Dual-infected, or pSMV-GFP/nuCFP-infected leaves of each soybean plant, were analyzed by RT-PCR. The progeny viruses were transferred three times from plant to plant by mechanical sap-inoculation. The numbered lanes indicate three individual plants tested in each passage. The arrowheads point at the RT-PCR products of the expected sizes.

To evaluate simultaneous expression of two recombinant proteins from the SMV dual vector in soybean, two fluorescence reporter genes, *gfp* and *cfp*, were cloned. In addition, to specifically visualize the expression of the resulting fluorescence proteins (i.e., GFP and CFP), CFP was expressed as a fusion protein tagged with the nuclear localization signal of SV40 T antigen (NLS). The *gfp* and NLS-tagged *cfp* genes were inserted into pSMV-Dual utilizing the cloning sites *Xba*I and *Sal*I, respectively, resulting in a construct designated as pSMV-GFP/nuCFP (Fig. 2B). Five micrograms of pSMV-GFP/nuCFP plasmid DNA were inoculated onto the primary leaves of soybean seedlings by direct rub-inoculation. At 15 dpi, typical mild mosaic symptoms appeared on the systemic leaves of the soybean plants inoculated with pSMV-GFP/nuCFP, similar to infection by the pSMV-Dual (data not shown). To verify whether the inoculated soybeans were infected systemically with the recombinant SMV, we extracted total RNA from upper uninoculated leaves at 15 dpi and subjected the extracts to RT-PCR using SMV-specific primers. The RT-PCR results confirmed that all of the inoculated soybean plants were infected systemically (data not shown). Next, systemic leaves of soybean plants inoculated with pSMV-GFP/nuCFP were subjected to confocal microscopy at 15 dpi to monitor fluorescent signals. As expected, the GFP signals were observed throughout the cytoplasm as well as in the nucleoplasm and the plasma membrane of the soybean cells, while the CFP signals were detected specifically in the nucleus (Fig. 2B), indicating that GFP and NLS-tagged CFP proteins were successfully expressed in the systemic leaves via the SMV-based dual vector. As a negative control, plants were inoculated with pSMV-Dual (empty vector) and no fluorescent signal was evident in the systemic leaves of the inoculated plants (Fig. 2B).

Few studies have been performed to the visualize subcellular distribution of cellular proteins in soybean cells mainly because of the unavailability of agroinfiltration in soybean leaves. In our confocal microscopy with high magnification, we clearly observed free GFP in the cytoplasm and the nuclear localization of NLS-tagged CFP in soybean cells. Thus, our approach will be useful to examine the subcellular localizations of host cellular proteins by expressing target proteins tagged with a fluorescence protein.

To test the stability of heterologous gene insertion in the SMV genome, the recombinant virus was transferred three times from plant to plant by mechanical sap-inoculation. Total RNA was isolated from each inoculated plant and analyzed for stable insertion of the *gfp* and *cfp* genes in the viral genome by RT-PCR using primer pairs spanning the gene insertion cassettes (Fig. 2C). Only amplicons with the predicted sizes were detected for viral genomes carrying both *gfp* and *cfp* genes (Fig. 2C). In addition, the fluorescence signals of GFP and CFP were readily detected in all soybean plants infected with pSMV-GFP/nuCFP or its progeny viruses through serial passages (data not shown). These results indicate that the dual-gene insertion in the viral genome was stably maintained during virus replication even after three serial passages.

### Application of the SMV-based dual vector for *in vivo* visualization of protein–protein interactions in soybean

Protein–protein interactions are basic cellular events in the control of many cellular processes. Thus, characterizing protein–protein interactions is essential for unraveling the biological functions of proteins and is becoming increasingly important in understanding various aspects of cell biology. Various approaches have been developed to examine protein–protein interactions *in vitro* and *in vivo*^13^,^14^,^15^,^16^. Among them, BiFC has been used as a convenient and powerful tool for identifying and visualizing protein–protein interactions in living cells^13^,^17^. Currently, BiFC, in association with agroinfiltration, has been used widely to examine protein–protein interactions and the subcellular localization of the interacting protein partner in various plant systems^13^. BiFC requires co-expression of two target proteins fused with the N- and C-terminal nonfunctional halves of a fluorescent protein. Only when the two target proteins interact can the N- and C-terminal YFP fragments be brought into close proximity to reconstitute functional YFP as a result of specific protein interactions. However, *in planta* application of BiFC analysis has not been performed in soybean because of a lack of efficient methods for synchronized expression of two recombinant proteins in soybean cells.

**Figure 3:**
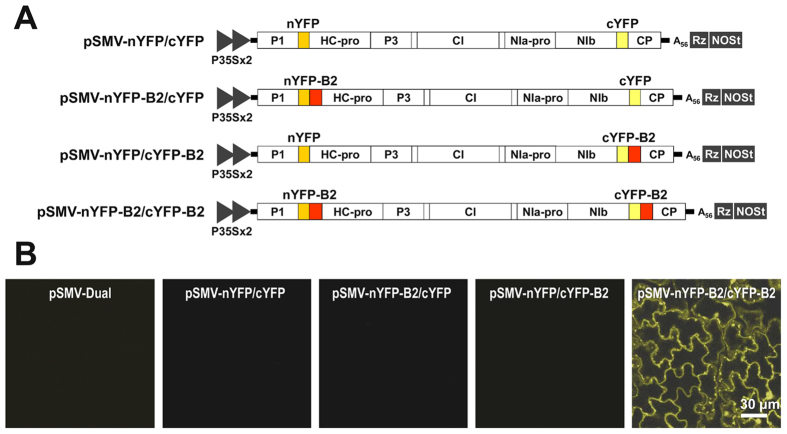
*In vivo* characterization of FHV B2 self-interaction in soybean cells by BiFC in combination with SMV-based dual-gene delivery. (**A**) Schematic representation of SMV recombinant constructs applied for BiFC assay to examine FHV B2 self-interaction. The open reading frames (ORFs) of nYFP, cYFP, nYFP-B2, and cYFP-B2 were in-frame inserted into the gene insertion cassettes of pSMV-Dual vectors for simultaneous expression. (**B**) *In vivo* visualization of FHV B2 self-interaction in soybean cells. Each SMV recombinant construct was mechanically inoculated into soybeans (cv. Lee74) as indicated at the top of each image. The reconstructed YFP signals were observed in the upper uninoculated leaves using confocal microscopy at 15 days post infiltration (dpi).

Because the SMV-based dual vector was used successfully to simultaneously express two recombinant proteins in a single cell and to visualize subcellular compartments emitting fluorescence signals using confocal microscopy, we sought to examine whether dual-gene delivery by the SMV-based dual vector could be applied for BiFC analysis of protein–protein interactions in soybean cells. A previous study showed *in vivo* self-interaction of protein B2 of flock house virus (FHV) by employing a BiFC assay in association with agroinfiltration in *Nicotiana benthamiana*^18^. Thus, we decided to test whether the B2 self-interaction could be detected in soybean cells when the same fusion recombinant B2 proteins are expressed by the SMV-based dual vector. To this end, we constructed four recombinant SMV constructs that express one of the following: (i) the N-terminal region (amino acids 1 to 156; nYFP)+the C-terminal region (amino acids 157 to 239; cYFP) of yellow fluorescent protein (YFP) (pSMV-nYFP/cYFP); (ii) nYFP-fused B2 + cYFP (pSMV-nYFP-B2/cYFP); (iii) nYFP + cYFP-fused B2; or (iv) nYFP-fused B2 + cYFP-fused B2 (Fig. 3A). Each construct was rub-inoculated on the leaves of soybean seedlings and the reconstructed YFP signals were monitored at 15 dpi using a confocal microscope. Strong fluorescence signals were observed in soybean cells when nYFP-B2 and cYFP-B2 were co-expressed by the SMV-based dual vector (Fig. 3A). However, the expression of other combinations employed as negative controls did not induce any detectable fluorescence signals, indicating that the fluorescence signals observed in the co-expression of nYFP-B2 and cYFP-B2 resulted from specific self-interaction of B2. The results suggest that our approach utilizing a viral dual vector can be a useful and efficient tool for *in vivo* characterization of protein–protein interactions in soybean and other plant systems.

### Application of the SMV-based dual vector for co-immunoprecipitation-based identification of cellular interacting protein partners

The identification of interacting protein partners in a given pathway often provides decisive clues to establish a hierarchical mechanism of a system biology. Among the various strategies, co-IP coupled with mass spectrometric analysis is one of the most popular techniques for identification of interacting protein partners^19^,^20^. Co-IP uses an antibody that specifically binds to a target protein to isolate this protein and its interacting partners from cellular lysates. Co-IP can be performed by ectopically expressing a recombinant protein tagged with a small epitope such as HA and Flag^21^. In this case, the recombinant protein can be immunoprecipitated by epitope-specific antibodies. The immunoprecipitated protein complexes then can be identified directly by mass spectrometric analyses, such as liquid chromatography coupled with tandem mass spectrometry (LC-MS/MS) and matrix-assisted laser desorption/ionization time-of-flight MS (MALDI-TOF MS)^20^,^21^.

Despite the recent abundance of soybean genomic data, only limited information is available on soybean protein–protein interaction networks when compared with other model plant systems. This is mainly due to difficulty of transient expression of recombinant proteins in soybean as mentioned above. The SMV-based dual vector is capable of expressing recombinant proteins at a high level because the recombinant proteins are synthesized as part of the viral polyprotein^7^,^8^. Thus, we sought to examine whether transient expression of a recombinant protein tagged with an epitope using the SMV-based vector is applicable for identification of cellular interacting protein partners by co-IP followed by mass spectrometric analysis.

The potyvirus HC-Pro is a multifunctional protein involved in crucial steps of virus infection^22^. HC-Pro has proteolytic activity to cleave at its carboxyl-terminus and is required not only for aphid transmission but also for long-distance systemic movement in plants, symptom expression, and suppression of RNA silencing. The multifunctional activities of HC-Pro may be regulated by interactions with other viral and cellular proteins. Indeed, direct interaction between potyvirus HC-Pro and CP mediates aphid transmission^23^,^24^. In addition, it has been shown that HC-Pro self-interacts to form oligomers, including dimers, tetramers, and hexamers^25^.

**Figure 4:**
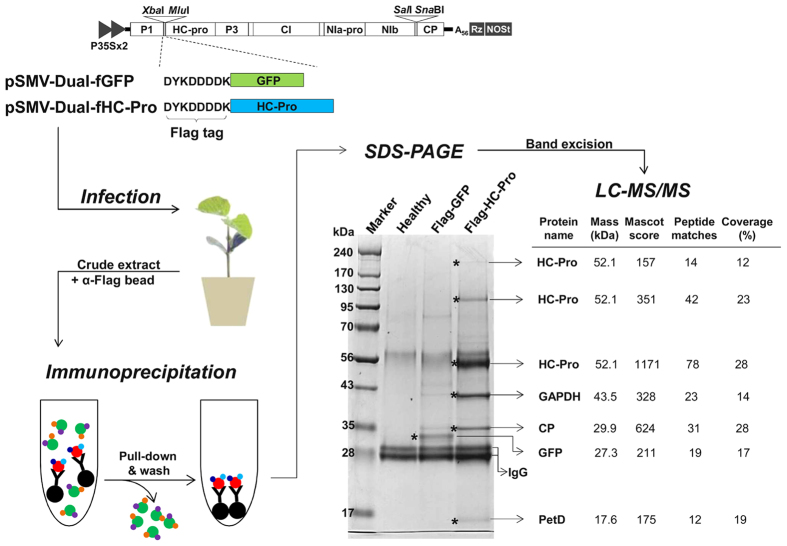
Workflow of the SMV-based gene delivery for identification of cellular interacting protein partners. Schematic representation of SMV recombinant constructs shows in-frame insertion of the Flag-tagged GFP and Flag-tagged HC-Pro into the P1/HC-Pro gene insertion cassette of pSMV-Dual vectors. Each SMV recombinant construct was rub-inoculated on the leaves of soybean seedlings. At 15 dpi, crude plant extracts prepared by homogenizing the upper symptomatic leaves were subjected to immunoprecipitation using anti-Flag antibody-conjugated agarose beads. The resulting co-immunoprecipitated products were analyzed by SDS-PAGE and the bands of interest (indicated by asterisks) were excised from the gel and subjected to LC-MS/MS analysis. The identified proteins and MS/MS spectral information are shown.

To confirm these known interactions by co-IP and to identify additional cellular interacting partner proteins, we decided to transiently express the SMV HC-Pro tagged with the Flag epitope (Asp-Tyr-Lys-Asp-Asp-Asp-Asp-Lys) at the N-terminus using the SMV-based dual vector. To this end, we generated a recombinant SMV construct, pSMV-Dual-fHC-Pro, that expresses Flag-tagged HC-Pro (Fig. 4). In parallel, an additional SMV construct, pSMV-Dual-fGFP, that expresses Flag-tagged GFP, was generated and used as a negative control in co-IP experiments. Each construct was rub-inoculated on the leaves of soybean seedlings. At 15 dpi, crude plant extracts were prepared by homogenizing the upper symptomatic leaves in the extraction buffer. After removing cell debris by centrifugation, the resulting extracts were incubated with anti-Flag antibody-conjugated agarose beads followed by precipitation by centrifugation. The resulting co-immunoprecipitated products were analyzed by sodium dodecyl sulfate-polyacrylamide gel electrophoresis (SDS-PAGE) and stained with Coomassie Blue (Fig. 4). After staining, the specific dominant bands in each lane were excised from the gel and subjected to LC-MS/MS analysis. The identified proteins and MS/MS spectral information are shown in Fig. 4. As expected, one dominant band in the Flag-GFP co-IP sample was identified as GFP. A total of six bands were analyzed in the Flag-HC-Pro co-IP sample. The bands at position 220, 110, and 55 kDa were identified as HC-Pro whereas the band at position 35 kDa was identified as SMV CP. Therefore, our co-IP approach confirmed the previous findings of the HC-Pro oligomerization and the HC-Pro–CP interaction. The identification of HC-Pro from the bands at positions 220, 110, and 55 kDa indicates tetrameric, dimeric, and monomeric forms of HC-Pro, respectively. The HC-Pro self-interaction is likely quite strong because the oligomeric forms were detected under the denaturing conditions of SDS-PAGE. In addition, we identified two novel cellular interacting partners of HC-Pro, glyceraldehyde-3-phosphate dehydrogenase (GAPDH) at position 43 kDa and cytochrome b6/f complex subunit IV (PetD) at position 17 kDa. GAPDH is an important enzyme that plays a pivotal role in energy metabolism^26^. Recent studies have shown that GAPDH has multiple functions in DNA replication/repair, RNA transport, apoptosis, oxidative stress, membrane fusion, and cytoskeleton assembly^27^,^28^,^29^,^30^. In addition, a few studies have suggested that GAPDH may play a role in RNA virus replication^31^,^32^,^33^. On the other hand, PetD is required for photosynthetic electron transport^34^. The involvement of PetD in virus infection cycle has not been studied yet. We are currently performing a separate study to further characterize the interactions between HC-Pro and either GAPDH or PetD and to examine the roles of GAPDH and PetD in HC-Pro functions and SMV infection.

The accumulation of a large amount of genetic information through high-throughput technologies has increased the need for rapid and simple analyses of gene function. Agroinfiltration as a transient gene expression approach has been used extensively for *in vivo* gene function analysis in *N. benthamiana* and several other plant species^1^,^2^. However, agroinfiltration is not equally successful in different plant species because the compatibility between the plant and bacterium varies^35^. In addition, the leaf architecture of some plant species including soybean is likely unsuitable for the application of agroinfiltration^36^.

Soybean is one of the most important crop plants because it serves as a major ingredient for a wide range of foods and beverage products. Considering the importance of soybean as a major food crop, it is highly desirable to identify and evaluate valuable traits to improve productivity, environmental and disease resistance, and the commercial value. The whole genome of the soybean has been sequenced^37^,^38^ and high-throughput transcriptome analyses have identified numerous genes specifically involved in various biological processes^39^,^40^,^41^,^42^,^43^. However, most of the identified soybean genes still remain uncharacterized because of the lack of a versatile transient expression method such as agroinfiltration in soybean.

The interest in using plant systems as biofactories for production of valuable proteins has led to the development of various plant virus-based vectors^8^,^44^. In addition, plant virus-based vectors provide attractive and powerful tools for rapid introduction of genes of interest and characterization of their functions in plants. In the present study, we developed the SMV-based dual-gene delivery vector to simultaneously express two genes in soybean. We also showed the possible applications of the SMV-based dual vector in visualizing and characterizing protein subcellular localization and protein–protein interaction at the cellular level (Figs 2 and 3). In addition, we demonstrated that our approach in combination with co-IP and mass spectrometric analysis is useful for identification of cellular interacting protein partners in soybean (Fig. 4). Recently, we have identified a number of genes that are expressed differentially in susceptible and resistant responses against SMV in soybean by high-throughput transcriptome analysis^43^. We are currently characterizing the gain-of-function effects of some of these genes by simultaneously expressing two different genes using the SMV-based dual vector to evaluate synergistic effects of the genes.

Potyviruses constitute the largest genus of plant viruses and adopt the same gene expression strategy of proteolytic processing of polyprotein precursors, making heterologous technologies broadly applicable for manipulation of potyvirus genomes^10^,^45^,^46^. So far, various potyviruses including tobacco etch virus (TEV), turnip mosaic virus (TuMV), and zucchini yellow mosaic virus (ZYMV) that have a broad host range have been engineered to express genes of interest^46^,^47^,^48^. Therefore, our approaches shown in this study with the SMV-dual vector can be applied to other potyvirus-host plant systems for simultaneous expression of multiple genes, visualization of protein subcellular localization and identification and characterization of protein-protein interactions in plants in which agroinfiltration is unsuccessful. We expect that our method will become a versatile and powerful tool for studying protein–protein interactions and for rapid analysis of gene function in various plant species.

## Materials and Methods

### Construction of pSMV-Dual vector

The SMV-based dual-gene delivery vector was constructed by engineering an additional gene insertion cassette between NIb and CP cistrons of pSMV-MCS^7^. The NIb region spanning from the *Pml*I site to the 3′ end of NIb was amplified using a primer pair (5′-GTCAGATGTTC**CACGTG**CCAAA-3′ and 5′-TAAAGATACGGACTC**TACGTAGTCGAC**TGATTGTAAGGACACTGATTCACAACA-3′, *Pml*I, *Sal*I, and *Sna*BI sites are shown in bold). The CP and 3′ untranslated region (from the 5′ end of CP to the second *Pml*I site) was amplified using a primer pair (5′-**GTCGACTACGTA**GAGTCCGTATCTTTACAGTCAGGTAAGGAGAAGGAAGGA-3′ and 5′-GTCACCTGTAATTCA**CACGTG**G-3′, *Pml*I, *Sal*I, and *Sna*BI sites are shown in bold, the nucleotide sequence for the NIa-Pro cleavage site is underlined). The two PCR fragments were joined by joint PCR as described previously^49^. The resulting joined fragment was digested with *Pml*I and inserted into pSMV-MCS, which was opened with *Pml*I. The resulting construct was named pSMVG7H-Dual. Next, introduction of the non-aphid-transmissible mutation, T310A, into HC-Pro was performed as follows. The HC-Pro region spanning from the 5′ terminus to the *Kpn*I site was amplified from pSMVdHC-HC_T310A_, which contains the non-aphid-transmissible mutation (T301A) in HC-Pro^12^, using a primer pair (5′-TAGA**ACGCGT**GAGTCTGTCTCGTTGCAGTCCCAAAATCCTGAAGCTCAGTT-3′ and 5′-CCAGCTTTAAGAACAT**GGTACC**-3′, *Mlu*I and *Kpn*I sites are shown in bold, the nucleotide sequence for NIa-Pro cleavage site is underlined). The resulting PCR fragment was digested with *Mlu*I and *Kpn*I and inserted into pSMVG7H-Dual, which was opened with *Mlu*I and *Kpn*I. This final construct, which was named pSMV-Dual, contains two gene insertion cassettes and a non-aphid-transmissible mutation.

### Insertion of heterologous genes into pSMV-Dual vector

The *gfp* gene was amplified using a primer pair harboring *Xba*I sites (5′-GC**TCTAGA**ATGGTGAGCAAGGGCGA-3′ and 5′-GC**TCTAGA**GAGGATCCCCTTGTACAG-3′, *Xba*I sites are shown in bold). The NLS-tagged *cfp* gene was amplified using a primer pair harboring *Sal*I sites (5′-ACGC**GTCGAC**ATGGTGAGCAAGGGCGAGGA-3′ and 5′-ACGC**GTCGAC**GACCTTTCTCTTCTTCTTTGGAG-3′, *Sal*I sites are shown in bold). The resulting amplicons were digested with *Xba*I and *Sal*I and cloned into the first and second gene insertion cassettes of pSMV-Dual, respectively, for simultaneous expression of the two genes. A similar cloning strategy was applied for cloning of the nYFP, cYFP, nYFP-fused B2, and cYFP-fused B2 genes into pSMV-Dual. The nYFP, cYFP, nYFP-fused B2, and cYFP-fused B2 genes were amplified from the PZPn-nYFP-B2 and PZPn-cYFP-B2 clones^18^ using appropriate primer pairs (the list of the primers is available on request) and cloned into pSMV-Dual utilizing the *Xba*I and *Sal*I sites in the gene insertion cassettes, accordingly. The Flag-tagged *gfp* and HC-Pro genes were amplified using the primer pairs (for Flag-tagged *gfp*, 5′-GC**TCTAGA**GACTACAAGGACGACGATGACAAGATGGTGAGCAAGGGCGAGG-3′ and 5′-GC**TCTAGA**GAGGATCCCCTTGTACAGCT-3′; for Flag-tagged HC-Pro, 5′-GC**TCTAGA**GACTACAAGGACGACGATGACAAGTCCCAAAATCCTGAAGCTCAGT-3′ and 5′-GC**TCTAGA**ACCAACTCTGTAGAATTTCATCTC-3′; *Xba*I sites are shown in bold, the nucleotide sequence for the Flag epitope is underlined). The resulting amplicons were digested with *Xba*I and cloned into pSMV-Dual, which was opened with *Xba*I.

### Plant growth and inoculation

Soybean plants were grown in a growth chamber at 25 °C under a 16/8-h photoperiod. Seedlings were selected for inoculation when the cotyledons were fully expanded. Plasmid DNAs of the SMV constructs were prepared using the Plasmid Maxi Kit (QIAGEN, Valencia, CA, USA). Each cDNA plasmid was rub-inoculated as described previously^7^. To detect virus accumulation in the inoculated and upper uninoculated leaves, RT-PCR was performed using an SMV-specific primer pair (5′-GATTGGAAGCATGGCGATTT-3′ and 5′-TTCACATACYTCATGCCGTCAA-3′). We evaluated the stability of the heterologous gene insertion in the recombinant progeny viruses as described here. Total RNA was isolated from each inoculated plant and analyzed for stable insertion of heterologous genes in the viral genome by RT-PCR using the primer pairs spanning the gene insertion cassettes (for the P1/HC-Pro gene cassette, 5′-GAATGGGAAGCTCGTTAACGC-3′ and 5′-GGAGGCATTTTATCAAACACCTT-3′; for the NIb/CP gene cassette, 5′-ATATTGCAGAGACAGCTTTGAGAA-3′ and 5′-TCCAACATTTACATCTTTGCTGCT-3′).

### Aphid transmission assay

The aphid transmission assay was performed as described previously^7^. In brief, aphids (*A. glycines*) were reared in controlled environment chambers on soybean. Aphids, previously starved for 2 h, were placed on soybean leaves showing SMV symptoms and allowed to probe for 10 min. Then, 15 aphids were transferred to new healthy soybean seedlings and allowed to feed for 24 h before being killed with an insecticide. Inoculated plants were maintained for 3 weeks and SMV infection was verified by RT-PCR.

### Confocal microscopy

Fluorescence signals emitted by GFP, CFP, and YFP in soybean leaves were visualized by confocal microscopy. At 15 dpi, upper uninoculated leaves were observed for emission of fluorescence using a Leica SP8 laser-scanning confocal microscope (Leica, Wetzlar, Germany) equipped with a specific laser/filter combination to detect CFP (excitation at 458 nm), GFP (excitation at 488 nm), and YFP (excitation at 514 nm).

### Immunoprecipitation assay

Total protein extracts were prepared from the systemic leaves of the soybean plants inoculated with pSMV-Dual-fGFP and pSMV-Dual-fHC-Pro. At 15 dpi, the leaves were homogenized in three volumes of protein extraction buffer (20 mM Tris–HCl at pH 7.5, 300 mM NaCl, 5 mM MgCl_2_, 5 mM dithiothreitol, 0.5% Triton X-100, proteinase inhibitor cocktail [Sigma, St Louis, MO, USA]). Cell debris was removed by centrifugation at 18,000 *g* for 20 min at 4 °C. The resulting supernatants were incubated with anti-Flag antibody conjugated agarose beads (Takara, Japan) for 16 h at 4 °C. The immunocomplexes were then precipitated by centrifugation for 1 min at 8,200 *g* and washed five times in 1 mL of the protein extraction buffer. The resulting samples were analyzed by 10% SDS-PAGE and stained with Coomassie blue. The Xpert prestained protein marker (GenDEPOT, Barker, TX, USA) was used as the molecular mass. After staining, bands of interest were excised from the gel and subjected to in-gel digestion followed by LC–MS/MS analysis as described previously^50^.

### Peptide Sequence Analysis by LC-MS/MS and Database Search

The entire LC-MS/MS procedure was performed at Yonsei Proteome Research Center (Seoul, South Korea). Briefly, LC was performed with an Easy n-LC 1000 system (Thermo Fisher Scientific, Rockford, IL, USA). A C18-nanobore column (150 mm × 0.1 mm, 3-μm pore size, Agilent) was used for peptide separation. LTQ-Orbitrap mass spectrometry (Thermo Fisher, San Jose, CA, USA) was used to identify and quantify peptides. Xcalibur (version 2.1, Thermo Fisher Scientific, Waltham, MA, USA) was used to generate peak lists. The peak lists were examined by searching the National Center for Biotechnology Information database using the MASCOT search engine (http://www.matrixscience.com, Matrix Science, Boston, MA, USA). The acquired data were compared to the whole database with search parameters set as follows: enzyme, trypsin; allowance of up to one missed cleavage peptide; mass tolerance ±0.5 Da and MS/MS tolerance ±0.5 Da; modifications of methionine oxidation and cysteine carbamidomethylation when appropriate, with auto hits allowed and only significant hits to be reported. The proteins were identified on the basis of two or more peptides whose ion scores exceeded the threshold, *P* < 0.05, which indicated the 95% confidence level for these matched peptides.

## Additional Information

**How to cite this article**: Seo, J.-K. *et al.* Engineering of soybean mosaic virus as a versatile tool for studying protein-protein interactions in soybean. *Sci. Rep.* **6**, 22436; doi: 10.1038/srep22436 (2016).

## Figures and Tables

**Table 1:** Dose response of soybean plants to pSMV-Dual plasmid DNA by rub-inoculation.

Amount of inoculated DNA (μg/plant)	Infectivity^a^			
	1	2	3	Total
10	5/5^b^	5/5	5/5	15/15
5	5/5	5/5	5/5	15/15
1	5/5	5/5	5/5	15/15
0	0/5	0/5	0/5	0/15

^a^Soybean seedlings (cv. Lee74) were rub-inoculated with the corresponding plasmids.

^b^Number of systemically infected plants/number of plants inoculated. Virus infection of the upper non-inoculated leaves was confirmed by RT-PCR using SMV-specific primers at 15 dpi.

**Table 2:** Abolishment of aphid transmissibility of pSMV-Dual.

SMV construct	Aphid transmission		
	Exp. 1	Exp. 2	Exp. 3
Mock	0/5*	0/5	0/5
pSMV-G7H	5/5	5/5	5/5
pSMV-MCS	5/5	5/5	5/5
pSMV-Dual	0/5	0/5	0/5

^*^Number of plants infected over number of plants inoculated by aphid-transmission. Virus infection was confirmed by RT-PCR using SMV-specific primers.
